# Microplastics and Wear-Particle Biology in Joint Arthroplasty: Converging Mechanisms, Clinical Relevance, and Future Biomaterials

**DOI:** 10.7759/cureus.117529

**Published:** 2026-10-06

**Authors:** Mohamed Elsayed Abdelhalim Hassan

**Affiliations:** 1 Department of Orthopaedic Surgery, King Fahad Hospital, Al Baha, SAU

**Keywords:** biomaterials, environmental exposure, joint arthroplasty, microplastics, oxidative stress, polyethylene, wear debris

## Abstract

Total joint arthroplasty effectively restores function in end-stage joint disease, yet particle-induced periprosthetic osteolysis and aseptic loosening remain major causes of implant failure. Emerging evidence shows that environmental microplastics can accumulate in human bone marrow and synovial tissues, and their physicochemical similarities to prosthetic wear particles suggest potentially shared mechanisms of peri-implant inflammation. This review examines potentially shared mechanisms underlying innate immune activation, macrophage polarization, inflammasome signaling, and osteoclastogenesis and evaluates their translational relevance to arthroplasty failure and future biomaterial development. The pathogenic cascade induced by prosthetic debris involves inflammasome activation, pyroptosis, and receptor activator of nuclear factor kappa-B ligand (RANKL)-mediated osteoclastogenesis, ultimately contributing to periprosthetic osteolysis. Concurrently, translational evidence demonstrates the presence and bioaccumulation of environmental microplastics within human musculoskeletal tissues. These environmental polymers may induce cellular stress responses, oxidative stress, and activation of inflammatory signaling pathways, including nuclear factor kappa B (NF-κB) and mitogen-activated protein kinase (MAPK) pathways, that overlap with mechanisms implicated in particle-induced osteolysis. However, there is insufficient evidence to establish that environmental microplastics independently increase the clinical incidence of aseptic loosening. Current preventive strategies focus on improving implant material properties and reducing the generation and biological activity of wear particles, including the use of highly cross-linked and antioxidant-stabilized polyethylene. Environmental microplastics and prosthetic wear debris may therefore share converging inflammatory and osteolytic mechanisms that compromise the peri-implant bone microenvironment. While direct clinical causality between environmental microplastics and arthroplasty failure remains unproven, this intersection represents an emerging area of investigation in orthopedic biomaterials, periprosthetic immunology, and osteolysis prevention.

## Introduction and background

Total joint arthroplasty is a highly successful orthopedic intervention that significantly improves mobility and reduces pain for patients with degenerative osteoarthritis and inflammatory arthropathies [1]. Despite continuous advancements in surgical techniques, implant geometry, and bearing surface tribology, the long-term survivorship of joint prostheses is consistently challenged by the phenomenon of aseptic loosening [2]. Recent comprehensive meta-analyses encompassing 20 studies and 520,789 participants have established a pooled global prevalence of aseptic loosening in primary total hip arthroplasty at 1.96% [2]. The etiopathogenesis of this failure mechanism is driven by a chronic, cell-mediated immunological response to particulate wear debris generated by inevitable mechanical articulation and micromotion at implant interfaces [1]. This pathology, known clinically as particle-associated periprosthetic osteolysis, is characterized by the robust recruitment of innate immune cells, the subsequent release of pro-inflammatory cytokines, and a catastrophic uncoupling of normal bone remodeling that heavily favors osteoclastic bone resorption [3].

Concurrently, a novel exogenous variable has emerged within the domain of human systemic toxicology: environmental microplastics and nanoplastics. The exponential global proliferation of synthetic polymers has led to their continuous fragmentation into microplastics (defined as particles with a longest dimension of less than 5 mm) and nanoplastics (defined as particles with a longest dimension of less than 0.1 µm) [4]. These ubiquitous synthetic particles heavily infiltrate the human food chain, atmospheric ecosystems, and water supplies [5]. Recent breakthroughs in advanced spectroscopic tissue analysis have conclusively documented the systemic dissemination of these environmental polymers in humans, mapping their bioaccumulation in the systemic circulation, highly vascularized parenchymal organs, and, crucially, the musculoskeletal system [6].

The biological intersection between joint arthroplasty and environmental microplastic exposure presents an emerging area of pathophysiological interest. Both implant-derived wear debris (primarily composed of ultra-high-molecular-weight polyethylene (UHMWPE)) and environmental microplastics (predominantly composed of polyethylene (PE), polystyrene (PS), and polypropylene (PP)) are non-degradable, hydrophobic particulate polymers [7]. The continuous, long-term exposure of the periprosthetic microenvironment to a dual burden of endogenous implant wear debris and systemically deposited exogenous environmental microplastics raises hypotheses regarding possible additive effects on macrophage activation and periprosthetic osteolysis, enhanced baseline macrophage activation, and accelerated periprosthetic osteolysis.

This narrative review critically evaluates the converging biology of environmental microplastics and prosthetic wear debris. By synthesizing the highest-level verified evidence, this report dissects the shared physicochemical determinants of innate immune sensing, macrophage polarization, and inflammasome activation. Furthermore, it explicitly distinguishes among proven clinical facts, current translational evidence, and mechanistic hypotheses, outlining the implications of these converging mechanisms for the clinical diagnosis of aseptic loosening, the engineering of next-generation antioxidant biomaterials, and the future research agenda needed to decode this complex, systemic, and local pathology.

## Review

This article is a narrative review. It synthesizes clinical, translational, and experimental evidence on the biological effects of environmental microplastics and prosthetic wear particles and their relevance to periprosthetic osteolysis and aseptic loosening after total joint arthroplasty. Because of the heterogeneity of the relevant literature, a narrative rather than systematic approach was chosen. The literature spans environmental science, biomaterials engineering, immunology, and clinical orthopedics, with widely varying study designs and outcome measures. The search and selection process is described below to maximize transparency and reproducibility.

A literature search was performed in PubMed/MEDLINE, Scopus, Web of Science, and Google Scholar for articles published between January 2000 and July 2026. Search terms combined concepts related to microplastics ("microplastic*", "nanoplastic*", "polyethylene particles", "polystyrene", "polypropylene"), prosthetic wear ("wear debris", "wear particles", "UHMWPE", "highly cross-linked polyethylene", "vitamin E polyethylene"), and relevant biological or clinical outcomes ("osteolysis", "aseptic loosening", "macrophage", "inflammasome", "NLRP3", "RANKL", "osteoclastogenesis", "synovium", "bone marrow") using Boolean operators.

Articles were eligible if they were published in English in peer-reviewed journals and reported one or more of the following: detection or quantification of microplastics in human musculoskeletal tissues or fluids; implant retrieval or periprosthetic tissue analyses; clinical outcomes of arthroplasty related to wear, osteolysis, or aseptic loosening; or animal or in vitro experiments examining particle-induced inflammation, macrophage responses, inflammasome activation, or osteoclastogenesis. For clinical outcome data, systematic reviews, meta-analyses, and randomized controlled trials were prioritized when available. Studies were excluded if they were conference abstracts without full text, non-English publications, editorials, or opinion pieces without original data, or if they addressed particle exposure without relevance to immune, musculoskeletal, or implant-related outcomes.

What are microplastics, and how do they enter the musculoskeletal system?

Microplastics are broadly categorized by origin into primary and secondary types. Primary microplastics are intentionally manufactured at a microscopic scale for commercial and industrial applications, such as cosmetic microbeads and pharmaceutical vectors [7]. Secondary microplastics, which constitute the vast majority of environmental pollution, are formed unintentionally through the physical, chemical, and biological degradation of larger plastic debris [7]. Human exposure to these synthetic polymers is relentless and occurs via oral ingestion of contaminated food and water, respiratory inhalation of airborne microfibers, and direct dermal contact [8].

Following initial entry into the gastrointestinal or respiratory tracts, microplastics and nanoplastics with sufficiently small hydrodynamic diameters can cross intact epithelial barriers and translocate into the systemic circulation [9]. This circulatory transport facilitates the extravasation and bioaccumulation of microplastics in highly vascularized secondary tissues. Emerging human evidence demonstrates that the skeletal microenvironment acts as a highly efficient, long-term physiological sink for these environmental pollutants [9]. The bone marrow compartment, characterized by an extensive, slow-flowing sinusoidal capillary network, allows for the systemic sequestration of circulating microplastics.

Recent state-of-the-art tissue analyses utilizing laser direct infrared (LD-IR) chemical imaging systems and pyrolysis gas chromatography-mass spectrometry have conclusively identified microplastics deeply embedded within human bone marrow. In a landmark human tissue analysis mapping microplastic distribution, 25 distinct polymer types were identified within the bone marrow compartment, exhibiting an average abundance of 19.72 particles/g of tissue. Crucially, the analysis revealed that 89.82% of these sequestered marrow particles measured less than 100 µm, strictly underscoring the size-dependent nature of systemic translocation and subsequent deep-tissue retention [10].

Beyond the medullary cavity, systemically circulating microplastics have been detected within human synovial joints. Recent surgical retrieval studies using Raman and micro-Fourier transform infrared spectroscopy have successfully identified microplastics in the synovial tissue and synovial fluid of patients undergoing total joint arthroplasty [6]. The accumulation of these non-resorbable particles within the avascular and slow-metabolizing connective tissues of the joint, such as hyaline cartilage and the menisci, suggests an overlooked mechanism of long-term physical burden that bypasses standard physiological clearance mechanisms [6].

The systemic distribution of microplastics into the musculoskeletal system significantly disrupts local homeostasis through multiple vectors. These include direct physical cellular damage, chemical toxicity mediated by leaching of plasticizers and absorption of environmental pollutants, and the direct induction of localized reactive oxygen species (ROS) [9]. While the mechanisms governing the initial deposition of these particles are becoming clearer, no verified higher-level evidence exists regarding the exact long-term kinetics of microplastic clearance versus permanent retention in human bone tissue [9]. The persistence of these non-resorbable polymers effectively generates a sustained, low-grade inflammatory stimulus within the bone-marrow niche and synovial fluid, establishing a volatile baseline ahead of any surgical intervention [9].

Prosthetic wear particles: established biology of particle disease

The biological response to implant-derived wear debris is a foundational pillar of orthopedic pathology, originally recognized and characterized by Willert and Semlitsch in the late 1970s [11]. Aseptic loosening is defined as the progressive failure of the implant-bone interface in the strict absence of microbial infection, driven almost exclusively by particle-associated periprosthetic osteolysis [1]. Artificial joint articulation, regardless of the specific bearing surface materials utilized, inevitably generates a continuous shedding of microparticulate and nanoparticulate debris [1]. In conventional metal-on-PE bearing combinations, billions of ultra-high-molecular-weight PE (UHMWPE) particles are shed annually into the surrounding periprosthetic capsular tissues [5].

Current international consensus statements and clinical guidelines establish that the diagnosis of aseptic loosening relies predominantly on serial radiographic evaluations [12]. Key radiographic indicators include progressive radiolucency at the bone-cement or bone-implant interface, endosteal scalloping, implant migration or subsidence, and frank focal osteolysis [12]. Notably, conventional radiography possesses a low diagnostic sensitivity in the early stages of disease; biological demineralization may remain radiographically occult until more than 30-50% of the local cancellous bone mass has been resorbed [13]. Diagnostic guidelines strictly mandate the comprehensive exclusion of periprosthetic joint infection (PJI) prior to confirming an aseptic etiology, using major and minor criteria, including synovial fluid polymorphonuclear neutrophil percentage (PMN%) and leukocyte esterase testing [14].

The pathogenesis of wear-particle disease is initiated by the generation of debris predominantly ranging from 0.1 to 10 µm [15]. Particles within this specific, highly reactive size range, particularly submicron PE debris (which exhibits an average diameter of approximately 530 nm in failed human implants), are aggressively phagocytosed by tissue-resident macrophages and recruited circulating monocytes [5]. Unlike degradable biological debris or necrotic host tissue, synthetic UHMWPE and metallic particles (e.g., cobalt-chromium or titanium alloys) cannot be enzymatically digested within the acidic environment of the macrophage phagolysosome [3].

This inability to degrade the engulfed synthetic material results in "frustrated phagocytosis." The macrophages fuse to form multinucleated foreign-body giant cells, initiating a self-amplifying positive feedback loop of chronic sterile inflammation within the neosynovial stroma, often referred to as the synovial-like interface membrane (SLIM) [11]. Activated macrophages within the SLIM actively secrete a dense cascade of pro-inflammatory cytokines, chemokines, and local growth factors, including tumor necrosis factor-alpha (TNF-α), interleukin-1 beta (IL-1β), and interleukin-6 (IL-6) [1].

This localized inflammatory cytokine storm radically alters bone metabolism at the implant interface. It simultaneously suppresses osteoblastic bone formation and aggressively stimulates osteoclastogenesis via the upregulation of receptor activator of nuclear factor kappa-B ligand (RANKL) by local fibroblasts, suppressed osteoblasts, and immune cells [16]. The net biological vector of this cellular interplay is the progressive, runaway osteoclastic resorption of the supporting peri-implant cancellous and cortical bone, which ultimately results in the catastrophic mechanical failure of the arthroplasty components [3]. While the macroscopic clinical manifestations of this cascade are well-documented, no verified higher-level evidence exists specifying the exact threshold volume of wear debris required to universally trigger this cascade in all human subjects, highlighting significant individual variations in immunological tolerance.

Shared physicochemical determinants of biological response

The biological reactivity and ultimate toxicity of both environmental microplastics and prosthetic wear debris are fundamentally dictated by shared physicochemical properties: specific particle size, polymer composition, surface topography, and high resistance to hydrolytic degradation. Quantitative identification of these particles in human tissue remains analytically challenging, as current methodologies struggle to distinguish endogenous prosthetic-derived PE nanoplastics from exogenously acquired environmental microplastics [17].

Particle size and geometry are the most critical determinants of cellular internalization and subsequent cytotoxicity. In the context of prosthetic biology, particles smaller than 1.7 ± 0.7 µm are strongly associated with significantly increased rates of clinical osteolysis and rapid macrophage death [3]. Symmetrically, human bone marrow analyses confirm that environmental microplastics are predominantly sequestered in the sub-100 µm range, with nanoplastics (<1 µm) possessing the highest inherent capacity to penetrate cellular membranes, translocate systemically, and accumulate intracellularly within the central nervous and musculoskeletal systems [6]. Furthermore, particle geometry strictly dictates immune tolerance; thick, irregular synthetic biomaterials with sharp, acute angles enhance foreign body reactions and provoke higher levels of mechanical cellular membrane damage compared to smooth, spherical configurations [18].

Regarding polymer composition, environmental microplastics consist largely of PE, PS, PP, and polycarbonate (PC) [6]. Prosthetic wear debris is overwhelmingly dominated by UHMWPE [19]. The stable carbon-backbone chemistry of these polymers renders them highly hydrophobic and virtually immune to enzymatic or hydrolytic degradation. However, both environmental microplastics and prosthetic PE are highly susceptible to oxidative degradation in vivo. In the joint space, mechanical articulation combined with exposure to synovial ROS causes severe chain scission and surface oxidation of the polymer, drastically increasing its bioreactivity [20]. Environmental microplastics undergo identical oxidative weathering through ultraviolet radiation and environmental exposure prior to ingestion by hosts, rendering their surfaces highly reactive upon entering the human body [9].

Upon entering physiological fluids - whether human synovial fluid or systemic blood plasma - both classes of particles rapidly adsorb host proteins, forming a dense biomolecular "corona." The surface functionality of these particles dramatically increases their capacity to bind endogenous proteins and exogenous toxins [21]. Microplastics act as highly mobile vectors, adsorbing endocrine-disrupting chemicals (e.g., polychlorinated biphenyls and perfluoroalkyl substances), trace organic compounds, and heavy metals [22]. In the context of joint arthroplasty, co-exposure to metallic nanoparticles (e.g., chromium ions released from hardware) and microplastics may produce additive or synergistic effects, as suggested by experimental models. In vitro translational models demonstrate that combined exposure to chromium nanoparticles and microplastics increased ROS and pro-inflammatory cytokine expression in local muscle and bone cells compared to either contaminant operating alone [23].

The transcriptional response of human joint tissues to these combined stimuli is consistent with a partially overlapping toxicity profile. Advanced transcriptomic analyses of human synovial tissue reveal that a mere three-fold increase in microplastic abundance directly corresponds to enhanced local cellular stress responses, specifically upregulating extensive heat shock protein signaling pathways [6]. Both particle types ultimately converge to act as sterile, particulate damage-associated molecular patterns (DAMPs) that can activate innate immune pathways.

Innate immune sensing, macrophage activation, inflammasome signaling, and osteoclastogenesis

The fundamental pathophysiological mechanism driving aseptic loosening involves the conversion of a mechanically stable, well-integrated implant interface into a highly inflammatory, osteolytic environment. The exact cellular signaling pathways engaged by prosthetic wear debris over the past three decades are now being identified in the rapidly evolving pathology of environmental microplastics, confirming a deep and dangerous mechanistic convergence.

Macrophages serve as the primary sentinels, orchestrating the biological response to particulate debris [24]. Upon exposure to either environmental microplastics or prosthetic wear particles, local tissue macrophages undergo profound phenotypic polarization. Wear debris and endotoxins present at the bone-implant interface strongly drive macrophages toward a classical, pro-inflammatory M1 phenotype, simultaneously suppressing the immunoregulatory and tissue-healing M2 phenotype essential for implant integration [25]. Microplastic exposure seamlessly replicates this polarization. In synovial tissue, microplastics alter the expression of specific regulatory microRNAs; for example, the upregulation of miR-155 in synovial macrophages heavily biases the cell toward the massive production of pro-inflammatory cytokines, directly aggravating rheumatoid and osteoarthritic joint pathology [26]. Additionally, the suppression of miR-132 and miR-21 further abrogates anti-inflammatory IL-10 production [26]. Prolonged M1 polarization induced by these non-degradable particles guarantees chronic inflammation and eventual osteolytic destruction [25].

The initial recognition of sterile polymer particles is mediated by pattern recognition receptors (PRRs), predominantly Toll-like receptors (TLRs), located on the macrophage surface. Both PE and metallic wear particles promote the robust expression of TLRs, which signal intracellularly through the myeloid differentiation primary response 88 (MyD88) adapter protein [25]. Activation of this axis triggers the nuclear factor kappa-light-chain-enhancer of activated B cells (NF-κB) and mitogen-activated protein kinase (MAPK) signaling cascades. Recent studies confirm that PS microplastics directly activate the same NF-κB/MAPK pathways in synovial fibroblasts, thereby promoting aggressive joint inflammation and cartilage destruction [27]. Furthermore, systemic microplastic exposure triggers massive oxidative stress, disrupting the NRF2/KEAP1 antioxidant signaling pathway and overwhelming the cell's redox homeostasis, which further amplifies NF-κB signaling [28].

The most critical converging mechanism identified in particle-induced osteolysis is the activation of the NOD-like receptor family, pyrin domain-containing 3 (NLRP3) inflammasome [29]. Phagocytosis of abrasive wear particles or microplastics physically destabilizes the macrophage lysosome, releasing active cathepsins and massive amounts of ROS directly into the cytosol [30]. This severe intracellular stress provides the required secondary activation signal for the assembly of the NLRP3 inflammasome complex.

Upon assembly, NLRP3 activates Caspase-1, which subsequently cleaves pro-IL-1β and pro-IL-18 into their active, highly inflammatory secretory forms [29]. Concurrently, Caspase-1 cleaves Gasdermin D (GSDMD), executing a highly inflammatory, lytic form of programmed cell death known as pyroptosis [31]. In vivo murine models unequivocally demonstrate that exposure to microplastics heavily exacerbates synovial cell pyroptosis via the same NLRP3 pathway, severely disrupting synovial tissue architecture and function [28].

The indispensable role of the NLRP3 inflammasome in particle-associated osteolysis has driven the exploration of targeted pharmacological inhibition. Translational evidence confirms that the pan-cathepsin inhibitor Ca-074Me and the specific NLRP3 inflammasome small-molecule inhibitor MCC950 effectively block wear debris-induced osteolysis in vitro and in pre-clinical animal models [29]. Despite these highly promising translational results, no verified higher-level evidence currently exists for the clinical efficacy, safety, or routine use of MCC950 or other targeted NLRP3 inhibitors in human patients with total joint arthroplasty.

The ultimate clinical consequence of this intense, particle-driven inflammatory cascade is the profound dysregulation of normal bone remodeling. The flood of IL-1β, TNF-α, and IL-6 generated by M1 macrophages and stressed synovial fibroblasts stimulates local osteoblasts and stromal cells to massively overexpress RANKL while simultaneously downregulating its protective decoy receptor, osteoprotegerin (OPG) [16]. The severely skewed RANKL/OPG ratio aggressively drives the differentiation and fusion of monocyte precursors into mature, multinucleated, bone-resorbing osteoclasts [24]. Both microplastic toxicity and prosthetic wear debris predictably culminate in this identical end-stage pathway: uncoupled, runaway osteoclastogenesis leading to catastrophic periprosthetic osteolysis [16].

Evidence in human arthroplasty tissue and current clinical relevance

The transition from in vitro mechanistic biology to definitive human clinical pathology requires rigorous, verified tissue analysis. Recently, groundbreaking surgical retrieval studies have conclusively documented the physical presence of environmental microplastics in primary human arthroplasty tissue, highlighting the insidious nature of this systemic exposure.

In a highly robust evaluation of human lower-limb joints, matched synovial samples were harvested from 45 patients undergoing primary total hip or total knee arthroplasty. Utilizing advanced micro-Fourier transform infrared spectroscopy and scanning electron microscopy, investigators definitively identified 343 distinct microplastics embedded within the human joint tissue [6]. The identified particles spanned nine common commercial polymer types, exhibiting a mean abundance of 5.24 ± 2.07 particles/g of human tissue (with abundances ranging from 1.16 to 10.77 particles/g). Notably, the abundance of microplastics proved anatomically variable, demonstrating significantly higher sequestration in the hip joint samples compared to the knee samples [6]. Demographic variables showed no clear correlation with microplastic abundance, indicating that accumulation is likely a universal phenomenon rather than one tied strictly to specific age or lifestyle demographics [6].

These environmental polymers are not biologically inert. Direct transcriptomic analysis of these matched human synovial samples revealed an apparent dose-response relationship: a three-fold increase in microplastic abundance statistically correlated with significantly enhanced local cellular stress responses, specifically triggering widespread heat shock protein reactions [6]. This confirms that the presence of synthetic polymers in the joint space, fully independent of macroscopic prosthetic wear, actively maintains local synovial stress and immune vigilance. Furthermore, LD-IR analyses of human bone marrow extracted during standard orthopedic procedures revealed the detection of 25 polymer types, with average concentrations of 19.72 particles/g [10].

Despite these translational findings, the direct clinical relevance of environmental microplastics in independently driving the failure of total joint arthroplasty remains highly complex and largely unproven. Current human biomonitoring methodologies struggle fundamentally to differentiate endogenously generated prosthetic nanoplastics (e.g., UHMWPE wear debris) from exogenously acquired environmental microplastics due to polymer similarities [17]. Importantly, polymer identity alone cannot reliably establish the source of a particle, because PE detected in periprosthetic or distant tissues may originate from either the implant or the environment. Several complementary features may help attribute source. Particle morphology and size distribution differ between sources: PE wear debris is predominantly submicron and globular or fibrillar, whereas environmental microplastics are typically more heterogeneous fragments, fibers, films, and beads spanning a wider size range. Oxidation state, measured for example as a carbonyl index by Fourier-transform infrared spectroscopy, may reflect sterilization history, cross-linking and thermal treatment, or environmental weathering. Additive profiles may also be informative: medical-grade UHMWPE is manufactured without the pigments, plasticizers, and processing additives common in commercial plastics, apart from deliberately blended vitamin E in VEPE. Chemical signatures obtained by pyrolysis-gas chromatography/mass spectrometry or Raman spectroscopy, and potentially stable isotope ratios, have been proposed as further discriminators, although their use in this setting remains largely unvalidated. Anatomical context also matters: particles concentrated in the periprosthetic membrane adjacent to a bearing surface are more likely implant-derived than those found in non-arthroplasty joints, distant tissues, or samples taken before implantation. Finally, rigorous procedural and laboratory blank controls are essential, because surgical drapes, gloves, and laboratory consumables are themselves potential sources of contamination. Where possible, future studies should combine these approaches with documentation of patient implant and environmental or occupational exposure history, as no single method is currently sufficient to attribute particle origin. While massive meta-analyses and large cohort studies confirm that the incidence of aseptic loosening correlates strongly with patient BMI (OR = 1.116, p < 0.001), male sex (OR = 1.232, p = 0.016), and specific implant parameters, no verified higher-level evidence currently proves that geographical or dietary exposure to environmental microplastics independently increases the absolute risk of clinical arthroplasty failure [2].

Currently, the clinical diagnosis of aseptic loosening remains strictly reliant on established radiographic guidelines and the careful algorithmic exclusion of PJI [12]. However, the confirmed, high-volume bioaccumulation of microplastics in the human bone-marrow compartment and synovial fluid introduces a permanent, low-grade inflammatory baseline. This systemic priming probably lowers the threshold for macrophage hyperactivation when prosthetic wear debris is subsequently introduced following joint replacement [17].

Future biomaterials and preventive strategies

Given the well-characterized adverse biological response to non-degradable particles, the primary strategy in the orthopedic community to prevent aseptic loosening has focused on engineering bearing surfaces that drastically reduce volumetric wear and strongly resist oxidative degradation [32]. The evolution from conventional UHMWPE to highly cross-linked PE (HXLPE) represents the most significant advance in mitigating particle-associated osteolysis over the last two decades.

Systematic reviews and meta-analyses of long-term randomized clinical trials (incorporating up to 15-year follow-ups on 2,539 hips) report lower wear and osteolysis rates with HXLPE in the evaluated cohorts in total hip arthroplasty. The transition to HXLPE liners was associated with lower rates of the clinical incidence of osteolysis. In younger, highly active patient cohorts, long-term osteolysis rates decreased from 25.4% with conventional PE to just 4.05% with HXLPE. Similar reductions were observed in older patient cohorts (dropping from 29.7% to 6.6%). Furthermore, the incidence of aseptic loosening decreased symmetrically regardless of whether the components were cemented or uncemented [33]. Table 1 compares the physicochemical and biological characteristics of environmental microplastics with prosthetic wear debris.

**Table 1:** Comparative physicochemical and biological properties of environmental microplastics vs. prosthetic wear debris PE, polyethylene; PP, polypropylene; PS, polystyrene; PET, polyethylene terephthalate; PVC, polyvinyl chloride; UHMWPE, ultra-high-molecular-weight polyethylene; PMMA, polymethyl methacrylate; HXLPE, highly cross-linked polyethylene; CoCr, cobalt-chromium; IL, interleukin; TNF-α, tumor necrosis factor-α; NLRP3, nucleotide-binding oligomerization domain, leucine-rich repeat and pyrin domain-containing protein 3; PAHs, polycyclic aromatic hydrocarbons; PCBs, polychlorinated biphenyls The table was originally compiled by the authors based on the collected literature.

Property	Environmental Microplastics	Prosthetic Wear Debris
Source [7]	Degradation of consumer plastics in the environment; ubiquitous exposure (ingestion/inhalation)	In vivo generation from implant bearing surfaces and cement; internal source post-arthroplasty
Composition [6,17]	Diverse polymers (PE, PP, PS, PET, PVC, etc.); many secondary additives	Mainly UHMWPE (PE) and PMMA; metal alloys (CoCr, Ti) and ceramics (Al₂O₃, ZrO₂) release particles
Size range [3,5]	<5 mm by definition; often 1-100 μm (fibers, fragments) in biological samples; nanoplastics (<1 μm) possible	Typically nanometers to tens of microns; UHMWPE ~0.1-1.0 μm (bioactive range); metal/ceramic debris often <0.5 μm
Shape & morphology [6,18]	Irregular fragments, fibers, spheres; surface weathering can roughen texture	Irregular flakes and granules; metallic debris often spherical or needle-like; highly irregular in retrievals
Surface chemistry [9,20]	Hydrophobic polymers; may carry adsorbed pollutants (PCBs, PAHs); inert	Pristine implant polymers or oxides; metal surfaces often oxidized; some surface charge but largely biologically inert
Biopersistence [7,8,19,20]	Highly persistent; minimal biodegradation once formed	Biostable polymers or metal oxides; no enzymatic degradation in body
Macrophage recognition [5]	Phagocytosed when sufficiently small; elicit oxidative stress/inflam. in vitro	Readily phagocytosed; strongly activate macrophages and NLRP3 inflammasome
Cytokine response [1,3,16,23]	Evidence mainly from in vitro models (e.g., elevated IL-6, TNF-α) with large doses; in vivo role unproven	Well-documented induction of TNF-α, IL-1β, IL-6; correlates with wear volume
Preventive measures [20,32,33]	Largely environmental (reduce plastic use); no direct strategies for joints	Use of HXLPE, ceramic bearings, antioxidant polymers to minimize wear

However, the irradiation process used to physically cross-link the PE matrix inherently generates trapped free radicals, leaving the polymer highly susceptible to progressive in vivo oxidation, which can slowly compromise long-term mechanical integrity [20]. To counteract this material vulnerability, modern biomaterial engineers have incorporated active antioxidant doping, specifically utilizing vitamin E (alpha-tocopherol) stabilized HXLPE (VEPE) [34]. Vitamin E actively quenches free radicals, protecting the polymer from oxidative degradation while maintaining the superior wear resistance conferred by cross-linking [19].

Recent rigorous meta-analyses evaluating VEPE in primary total hip and knee arthroplasty provide robust evidence of its tribological efficacy. In vivo radiographic assessments demonstrate that vitamin E stabilization results in exceptionally low volumetric wear rates, generally yielding femoral head penetration rates of less than 0.05 mm/year [35]. Compared with conventional PE, VEPE demonstrates significantly less femoral head penetration at two and five years postoperatively [19]. Retrieval analyses confirm excellent resistance to oxidation with no reported osteolysis or adverse clinical events directly attributable to the vitamin E incorporation itself [20].

Despite these clear tribological advantages, meta-analyses incorporating thousands of knees (e.g., 2,285 knees analyzed in recent systematic reviews) currently indicate no robust evidence of a clinically observable benefit of VEPE over standard HXLPE in total revision rates or functional scores at short- to mid-term follow-up [20]. The relatively low base rate of aseptic loosening with standard HXLPE necessitates decades of follow-up to prove superiority in survivorship mathematically [36]. Nevertheless, by drastically minimizing the absolute volume of particulate debris generated, lower wear rates with VEPE would be expected to reduce particulate burden and may thereby limit exposure to the periprosthetic macrophages, thereby suppressing the initial trigger for TLR activation, NLRP3 inflammasome assembly, and subsequent clinical osteolysis. Table 2 shows a summary of the highest-level verified evidence identified to date.

**Table 2:** Summary of highest-level verified evidence identified to date HXLPE, highly cross-linked polyethylene; VEPE, vitamin E-stabilized polyethylene; NLRP3, nucleotide-binding oligomerization domain, leucine-rich repeat and pyrin domain-containing protein 3; PJI, periprosthetic joint infection The table was originally compiled by the authors based on the collected literature.

Clinical Domain	Key Verified Finding	Reference(s)
Diagnosis of Aseptic Loosening	Relies primarily on serial radiographs (progressive radiolucency, osteolysis); strict exclusion of PJI required.	[12]
Aseptic Loosening Risk/Prevalence	Pooled global prevalence of aseptic loosening in primary THA is 1.96% (n = 520,789).	[2]
HXLPE Clinical Outcomes	HXLPE reduces osteolysis incidence from 25.4% to 4.05% in young patients over 15 years.	[33]
Vitamin E PE (VEPE) Efficacy	VEPE achieves head penetration < 0.05 mm/yr; no proven long-term revision superiority over standard HXLPE yet.	[19]
Microplastics in Human Joints	Identified in lower limb synovium (mean 5.24 particles/g); correlates 3-fold with heat shock protein expression.	[6]
Microplastics in Bone Marrow	Deep embedding of 25 polymers in human bone marrow (19.72 particles/g; 89.8% < 100 µm).	[10]
Inflammasome Inhibition	MCC950 (NLRP3 inhibitor) actively blocks particle-induced macrophage pyroptosis and osteoclastogenesis.	[29]

Evidence gaps and research agenda

While the convergence of environmental microplastic toxicity and prosthetic wear biology is mechanistically compelling, significant and substantial gaps in the evidence hierarchy remain unaddressed.

First, analytical resolution within tissue retrieval studies must be vastly improved. Current human tissue retrieval studies lack the polymer-specific resolution and morphological detail needed to distinguish ingested environmental microplastics from internal prosthetic nanodebris reliably [17]. Future studies require the mandatory integration of advanced spectroscopic techniques, such as LD-IR and high-resolution Raman spectroscopy, to distinctively fingerprint environmental versus implant-derived polymers within the periprosthetic SLIM [10].

Second, the clinical and epidemiological impact of environmental microplastics on arthroplasty survivorship remains entirely hypothetical. While extensive meta-analyses have rigorously quantified the risk factors for aseptic loosening (e.g., BMI, implant design, male sex), no large-scale longitudinal cohort studies have successfully mapped environmental microplastic exposure to joint replacement outcomes [2]. Epidemiological investigations utilizing validated biomonitoring of systemic microplastic loads are essential to determine if high environmental exposure genuinely accelerates prosthetic loosening [22]. Without this data, no verified higher-level evidence is available to link environmental plastics to implant failure rates.

Third, while the NLRP3 inflammasome is widely recognized as the critical intracellular nexus for particle-induced osteolysis, clinical translation of targeted pharmacology (e.g., MCC950 or Ca-074Me) remains stagnant [30]. Future randomized controlled trials must evaluate whether perioperative or targeted local delivery of inflammasome inhibitors can safely attenuate wear-induced osteolysis in humans without causing profound, unacceptable immunosuppression or impairing initial osteointegration.

## Conclusions

Environmental microplastics and prosthetic wear debris share key inflammatory pathways, including macrophage activation, oxidative stress, NLRP3 inflammasome signaling, and RANKL-mediated osteoclastogenesis, which contribute to periprosthetic bone loss. Emerging evidence demonstrates the bioaccumulation of environmental microplastics within human bone marrow and synovial tissues; however, a direct clinical link between microplastic exposure and aseptic implant loosening remains unproven. Advances in biomaterials, particularly highly cross-linked vitamin E-stabilized PE, have substantially reduced wear-particle generation and osteolysis. Future research should focus on elucidating the clinical significance of environmental microplastics and on developing strategies to further enhance implant longevity amid increasing global microplastic exposure.
